# Adjustment of Synchronization Stability of Dynamic Brain-Networks Based on Feature Fusion

**DOI:** 10.3389/fnhum.2019.00098

**Published:** 2019-04-02

**Authors:** Haifang Li, Rong Yao, Xiaoluan Xia, Guimei Yin, Hongxia Deng, Pengfei Yang

**Affiliations:** College of Information and Computer, Taiyuan University of Technology, Taiyuan, China

**Keywords:** EEG, working memory, EEG dynamic brain network, brain network synchronization stability, brain network synchronization adjustment and control

## Abstract

When the brain is active, the neural activities of different regions are integrated on various spatial and temporal scales; this is termed the synchronization phenomenon in neurobiological theory. This synchronicity is also the main underlying mechanism for information integration and processing in the brain. Clinical medicine has found that some of the neurological diseases that are difficult to cure have deficiencies or abnormalities in the whole or local integration processes of the brain. By studying the synchronization capabilities of the brain-network, we can intensively describe and characterize both the state of the interactions between brain regions and their differences between people with a mental illness and a set of controls by measuring the rapid changes in brain activity in patients with psychiatric disorders and the strength and integrity of their entire brain network. This is significant for the study of mental illness. Because static brain network connection methods are unable to assess the dynamic interactions within the brain, we introduced the concepts of dynamics and variability in a constructed EEG brain functional network based on dynamic connections, and used it to analyze the variability in the time characteristics of the EEG functional network. We used the spectral features of the brain network to extract its synchronization features and used the synchronization features to describe the process of change and the differences in the brain network's synchronization ability between a group of patients and healthy controls during a working memory task. We propose a method based on the fusion of traditional features and spectral features to achieve an adjustment of the patient's brain network synchronization ability, so that its synchronization ability becomes consistent with that of healthy controls, theoretically achieving the purpose of the treatment of the diseases. Studying the stability of brain network synchronization can provide new insights into the pathogenic mechanism and cure of mental diseases and has a wide range of potential applications.

## Introduction

The brain is a complex system that exhibits various subsystems on different spatial and temporal scales. These subsystems are recurrent networks, that is, very large clusters of neurons that repeatedly interact with each other. Individual neurons are microscopic and change at a different time rate than macroscopic neural populations. After Babloyantz et al. (1986) first used nonlinear dynamics theory to study EEG signals in 1985, research on EEG signals rapidly entered the era of nonlinear dynamics. Various theories and methods of nonlinear dynamics have opened up new possibilities for analyzing EEG data. Eliasmith et al. (2012) presented a 2.5 million neuron model of the brain (called “Spaun”) that bridged this gap by exhibiting many different behaviors. The model is presented by only visual image sequences, and it draws all its responses with a physically modeled arm. Although simplified, the model captures many aspects of neuroanatomy, neurophysiology, and psychological behavior.

Hutt (2010) studied the main characteristics of a single neuron and its interactions by establishing a standard mathematical model and applied the model to explain experimental results from the delayed feedback system of weak electric fish and from electroencephalography (EEG). Liley et al. (2002) used nonlinear differential equations based on the human brain's physiological structure and medical anatomy to define a mathematical model of brain neuron clusters in states of both excitement and inhibition. With the establishment of neuron models, neuroscientists have conducted extensive and in-depth studies on neural network dynamics using various neuronal models to try to reveal the hidden secrets of the brain (Stam et al., 2007; Liu et al., 2008, 2014; De Han et al., 2009; Sun et al., 2009; Bartolomei et al., 2010; Skidmore et al., 2011).

Currently, many studies (Zhao et al., 2008; Qun, 2009; Gao et al., 2014b; Ruizhen et al., 2017) have shown that when the brain is active, the neural activities of different regions are integrated on a variety of spatial and temporal scales; this is known as the synchronization phenomenon in neurobiological theory. Synchronization is the basic mechanism for information integration and processing. Clinical medicine studies have shown that some of the neurological diseases that are difficult to cure have deficiencies or abnormalities in the whole or local integration process of the brain. Scientists have discovered a variety of synchronous behaviors in the neuronal system. The results of these studies show that the synchronization behavior of neuronal firing not only affects daily learning, brain memory, calculation, and motor control but can also be used to explain some neurological diseases such as epilepsy and Parkinson's disease.

The human brain is a complex network. Synchronization capability is an important indicator of complex networks. Therefore, brain network synchronization research has gradually attracted the attention of brain scientists and has made great advances. For example, Ma et al. (2014) and Hongli et al. (2013) found that the synchronization of the brain network of Alzheimer's patients was lower than that of a control group. Hou et al. (Dong et al., 2014; Feng-Zhen et al., 2014) analyzed the brain network of epilepsy patients using the network connectivity index to understand whether the brain network of patients with epilepsy is different from a normal brain network, and also investigated the brain electrical signal synchronization of patients with cerebral infarction. Rosário et al. (2015) proposed a new brain network edge association method that involves motif synchronization, primarily by calculating the number of occurrences of certain patterns between any two time-series to provide information about the degree and direction of synchronization between two nodes in the network. Sakkalis et al. (2013) used amplitude square coherence, phase synchronization estimation, and robust nonlinear state space generalized synchronization assessment methods to calculate the synchrony between all the pairs of channels in alcohol addiction patients. The experimental results showed that, during a rehearsal procedure, the alcohol addiction patients showed a loss of synchrony and an impaired lateralization of the brain activity.

Although previous studies have used synchrony to study neurodegenerative diseases, most of the current studies about the differences in brain function between patients with mental disorders and normal subjects investigated traditional features of brain network properties (node degree, mean-clustering-coefficient, global-efficiency, small-world attributes, etc.) (Micheloyannis et al., 2006; Zhang et al., 2013a,b, 2015; Müller et al., 2018). Researching these traditional features can clearly aid in understanding the topological characteristics of the brain network, but these features do not fully reflect the structure of the brain network. As a result, clinicians cannot find a unique and effective index for determining the specific diagnosis that a subject should receive. The spectral properties of complex networks (Li and Zhang, 1997; Xiao, 2012; Sato and Iwai, 2014; Liu and Shen, 2017) can provide a comprehensive measure of the global structure of the network. Any change in a local attribute feature is reflected in changes in the spectrum.

Therefore, to find more significant indicators of the differences between mental patients and healthy controls, we built a brain network based on complex network theory, used the spectral features of the brain network to identify the synchronization characteristics, and used the synchronous features to characterize the patients and the healthy controls. Thus, we studied the process by which the brain's synchronization ability changed during the working memory process and its difference between the two groups. We also proposed a method based on fusing traditional features and spectral features to adjust the synchronization ability of the brain networks of patients so that their synchronization ability will be consistent with those of healthy controls. Theoretically we can achieve the goal of treating diseases. Studying brain network synchronization can help to more clearly explain the dynamic process of the collective behavior of a large number of nodes in a complex brain network and may be able to prevent the harm that comes from some types of synchronizations. Thus, this research may provide a new direction for studying the pathological mechanisms of brain diseases. The brain network mechanisms of healthy controls and patients have very important practical significance and academic value.

The paper is organized as follows: Section EEG Dataset Description and Preprocessing briefly describes the dataset of EEG signals employed in our research. Section Methods presents information about the methods used in this study, including constructing the brain-network, extracting synchronization features, and synchronizing optimization algorithms. Section Experimental Results and Analysis provides the experiments undertaken in the framework of the study, the experimental procedures used, and the experimental results obtained. Finally, section Limitations describes the conclusions derived from the study and some thoughts, with regard to future work.

## EEG Dataset Description and Preprocessing

### EEG Dataset Description

**Figure 1:**
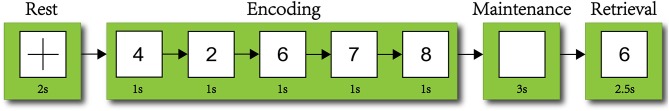
Short-term memory scanning task (SMTS) paradigm. Subjects needed to remember the numbers appearing on the screen during the encoding stage and recall these during the maintenance stage. Finally, in the retrieval stage, the subjects were required to determine whether the number had appeared by searching their memory.

The dataset used in this work was task state EEG data. The experimental paradigm used the modified Sternberg's SMST (Manoach et al., 1999) (short-term memory scanning task) paradigm (see Figure 1) The dataset included 34 psychiatric (in this study we used schizophrenic) patients and 34 healthy people (controls), none of whom had any record of drug abuse or diagnosis of neuropsychiatric disease in the past 6 months. The age range of the patient group was 20–51 years old, and the average age was (40.1 ± 11.1) years old; the healthy control group age range was 21–58 years old, and the average age was (37.1 ± 13.8) years old. Age, sex, and education level did not differ significantly between the two groups. All the members of both groups had normal vision or corrected visual acuity, had no color disturbance, and were right-handed (Zhao et al., 2011).

### Data Preprocessing

Data collection was completed at a hospital psychiatry research center on a NeuroScan 64-lead EEG acquisition device. The sampling frequency was 500 Hz, the impedance was kept below 5 kΩ, the ground electrode was AFz, and the reference electrode was physically connected to the left and right mastoids. The vertical electro-oculogram recording was from electrodes placed above and below the left eye, and the horizontal electro-oculogram recording was from electrodes placed on the right eyelid margin (Stam et al., 2007).

**Figure 2:**
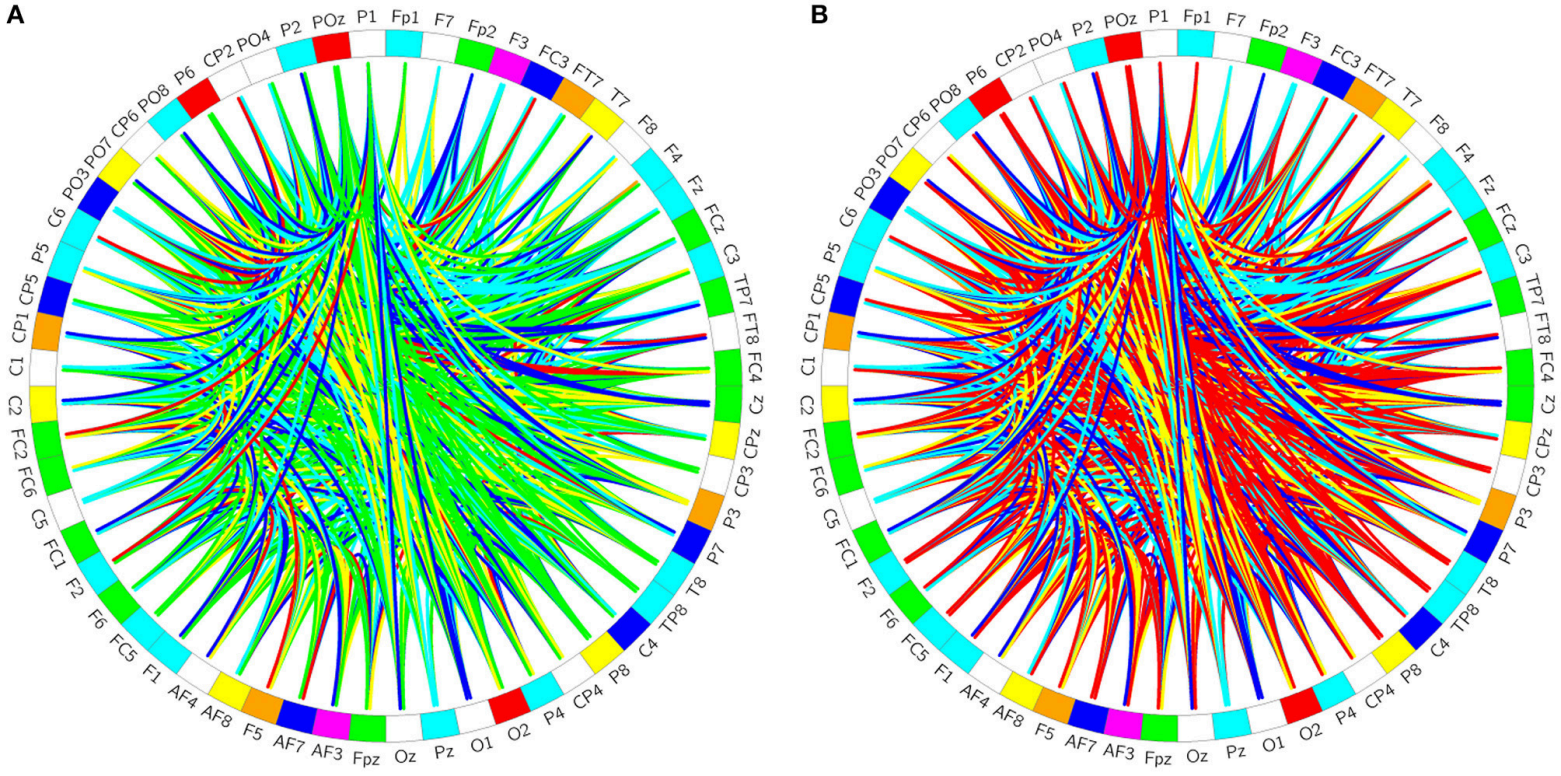
Brain-network. **(A)** Represents the healthy controls, **(B)** represents the patients.

The preprocessing was performed on the EEGLab (https://sccn.ucsd.edu/eeglab/download.php) platform on Matlab, which converted the reference data of the original data, removing the electrooculogram, filtering, segmenting, and removing artifacts to yield noiseless and clean EEG data. An average reference electrode was selected as the reference electrode, and the low-pass and high-pass noise were removed by filtering in the range of 0.5–50 Hz. The ocular electrical artifacts in the data were removed using the negative entropy-based FastICA method (Joyce et al., 2010; Jiaqing et al., 2018). Compared with the traditional blind source separation algorithm, this method does not require the ocular electrical signal as the reference electrode, avoiding the mixing of new noise during the EE signal acquisition process and reducing the collection workload. In addition, volume conduction can affect the output of synchronization measures when using EEG signals, because EEG is bipolar by nature. This means that EEG signals are composed of a difference between an electrode of interest and a reference (Guevara et al., 2005; Peraza et al., 2012). We used surface Laplacian transform methods (Matlab toolbox CSD) (Kayser and Tenke, 2006a,b) to eliminate the mixing effect of volume conduction. During the data acquisition process the data were labeled as S1–S10, in which S1–S5 represented the encoding phases, S6–S8 represented the maintenance phases, and S9–S10 represented the retrieval phases (see Figure 2). The data were filtered and retained by θ (4–7 Hz), α (7–14 Hz), β1 (14–20 Hz), β2 (for 20–30 Hz), and γ (30–40 Hz) signals in five frequency bands. From these data, 20 segments were selected from the different frequency bands in the different stages, and each was spliced. Duration of the EEG trajectory in the encoding, maintenance, and retrieval phases were 100 s, 60 s, and 50 s, respectively.

## Methods

### Unweighted Complex Dynamic Brain-Networks Construction

In the experiment, 60 scalp electrode channels were selected as the nodes of the brain network. The phase-locking value (PLV) coherence function was chosen for the edges of the network. This function was used to calculate the correlation between two electrode channels to form a 60 × 60 PLV correlation matrix. PLV can separate phase and amplitude components. Because EEG data can be affected by transient amplitude changes such as eye movements, PLV is quite suitable for this data. This paper uses a wavelet transform to extract phase information. The short time Fourier transform (STFT) method uses a sliding window to intercept the signal and performs a Fourier transform on the signal in the window to obtain the spectrum formula of the signal at any time (1), where STFT of *f(t)*: computed for each window centered at *t* = *t*′; *t*′ is the time parameter; μ is the frequency parameter; *f(t)* is the signal to be analyzed; *W*(*t*−*t*′) is the windowing function.

(1) $$\begin{array}{l} STFT\left(t^{\prime },\mu \right)=\int f\left(t\right)W\left(t-t^{\prime }\right)e^{-j2\pi \mu t}dt \end{array}$$

Specifically, the PLV definition (Xu et al., 2014) is Equation (2), where N represents the number of brain network nodes (In this paper *N* = 60); Δ φ_*n*_(*t*) = φ_*x*_(*t*)−φ_*y*_(*t*) represents the phase difference between the two channel time-frequency points t; and PLV ϵ [0,1].

(2) $$\begin{array}{l} PLV\left(t\right)=\frac{1}{N}|\overset{N}{\underset{n=1}{\sum }}exp\left(j\left(△\Phi_{n}\left(t\right)\right)\right)| \end{array}$$

The study of dynamic functional networks or time-varying brain function networks is an emerging field in brain function connection research. Its purpose is to study the dynamic nature or variability of functional connections over time. It has been applied to the analysis and diagnosis of brain diseases in fMRI and has in EEG signal analysis. Common methods for studying time-varying functional connections include important transfer point detection, time-frequency decomposition methods, and time window methods (Rosário et al., 2015). Of these, the sliding time window method is currently the most widely used. Existing research found that the brain network clearly shows time-variability and dynamics even over a short period of time and that the size of the time window is related to changes in the topological properties of the brain network (Sakkalis et al., 2013). The time window selected for the PLV cannot be too large. If it is too large, the signal may not be reasonably stable during this time period. Therefore, referring to existing research in a PLV phase synchronicity measurement study (Yi et al., 2014) and existing related research (Gysels and Celka, 2004; Gao et al., 2014a; Bola et al., 2015), we selected a sliding time window (step size of 0.04 s) in the range of 0.04–0.48 s. To determine the size of the PLV sliding time window, the classification accuracy of the network attributes was calculated within a given range of 0.04–0.48 s. The classification accuracy between the groups was determined. The final time window was determined to be 0.12 s.

The experiment was related to previous research on the small-world characteristics of the human brain (Guo et al., 2013). The sparsity range chosen was 30 to 40% with a step size of 2%; the network was constructed separately for each of the 20 trials. The networks for the 34 patients and 34 healthy controls were constructed separately for the encoding, maintenance, and retrieval stages and for the alpha and theta bands. A total of 48,960 brain networks were constructed.

### Brain-Network Feature Extraction

The traditional features of brain networks are usually attributes of the network and include global attributes and local attributes (Guo et al., 2013). Our experiment calculated four local attributes, which were degree, inference, clustering coefficient, and local efficiency, and six global attributes, which were global efficiency, modularity index, positive and negative matching degree, feature path length, average clustering coefficient, and average local efficiency. The feature extraction in this research included two stages: The first stage extracted the distinctive features from the traditional features; the second stage extracted the features of the brain network spectrum; and spectral feature calculations provided the synchronization features.

The first stage:

- Extraction of significant differences from global features

We compared the global attribute values between the patient group and the healthy controls in the same frequency band and at the same sparsity in the same stage. We used the Kolmogorov-Smirnov (KS) test (*P* < 0.05) to indicate that the node difference between the patients' brain networks and those of the healthy controls was significant. A significant difference attribute was put into a support vector machine (SVM) classifier as a feature. Based on an analysis of the classification result, a global attribute with a significant difference at a specific stage and a specific frequency band was selected.

- Extraction of significant differences and nodes from local attributes

To characterize the overall level of a property Y over a given sparsity range (Guo et al., 2013; Hao, 2013), these two papers from our research group used the area under the curve (AUC) to characterize the value of the entire sparsity range *Y*^*AUC*^ in the selected sparsity range. Its definition is shown in formula (3), where ΔS represents the space between the sparse upper bound Sn and the lower bound S1 span, which is the step size for the change in sparseness. In this study, the upper bound Sn was 40%, the next S1 was 30%, and the step size ΔS was 2%.

(3) $$\begin{array}{l} Y^{AUC}=\overset{n-1}{\underset{k=1}{\sum }}\left[Y\left(S_{k}\right)+Y\left(S_{k-1}\right)\right]\times \Delta S/2 \end{array}$$

In our current experiment, the sparsity range was fused by calculating the AUC. Throughout the entire sparsity range, we identified the nodes that had significant differences between the patients and the healthy controls. We tested the AUC value of each subject's local attribute value in the sparsity range at a certain stage and a certain frequency band. Then, we selected the local attribute AUC value splicing of the significant difference node as a feature to classify, and obtained locally significant attributes and significant nodes that differed significantly between the patients and healthy controls.

The second stage extracted the brain network spectrum characteristics and calculated the brain network synchronization characteristics according to section Synchronization Criteria. The spectral features of a network generally refer to the set of all the eigenvalues of the Laplacian matrix.

### Synchronization Criteria

Network synchronization is a very common and important non-linear phenomenon. There are many different types of network synchronization, such as common constant synchronization, phase synchronization, generalized synchronization, etc. Identical synchronization is defined as:

Definition 1:

Let *x*_*i*_(*t, X*_0_) be a solution of the complex dynamic network

(4) $$\begin{array}{l} ẋ=f\left(x_{i}\right)+g_{i}\left(x_{1},x_{2},x_{3},\ldots x_{N}\right),\;i=1,2,\ldots ,N \end{array}$$

where $\;X_{0}={\left({\left(x_{1}^{0}\right)}^{T},{\left(x_{2}^{0}\right)}^{T},\ldots ,{\left(x_{N}^{0}\right)}^{T},\right)}^{T}\in R^{N^{*}N},f:D\to R^{n}$ and $g_{i}:D\times D\to R^{n}\left(\;i=1,2,\ldots ,N\right)$ are all continuously differentiable, *D* ⊆ *R*^*n*^,and meet *g*(*x*_1_, *x*_2_, …, *x*_*n*_) = 0. There is any non-empty open set *C* ⊆ *F* in the domain, which can make any *x*_*i*_(*t, X*_0_) ∈ *F* and

$$\begin{array}{l} \lim_{t\to \infty }∥x_{i}\left(t,X_{0}\right)-s_{i}\left(t,X_{0}\right)∥=0\;i=1,2,\ldots ,N \end{array}$$

for any $x_{i}^{0}\in C,i=1,2,\ldots ,N\;$ and *t* ≥ 0, *i* = 1, 2, …, *N*, where *s*_*i*_(*t, X*_0_) is an effective solution space of equation ẋ = *f*(*x*), and *X*_0_ ∈ *F*, then the complex dynamics network can reach the identity synchronous steady state, and *C*×… × *C* is called the synchronous area of the complex dynamic network.

Identical synchronization is a common phenomenon of network synchronization, which shows that all nodes in the network are in the same state at a particular time point. In Definition 1, *s*(*t, X*_0_) is the synchronous steady state of the network, and *x*_1_ = *x*_2_ = … = *x*_*N*_ is the synchronization manifold of the network state space; that is, each physical oscillator tends to be in a described state when a network is synchronized.

Definition 2:

In 1998, Pecora and Carroll (Pecora and Carroll, 1990; Kashtan and Alon, 2005) studied the stability of the synchronization of linear coupled networks and developed the main stability function discrimination method. In 2002, Wang and Chen (Lü and Guanrong, 2005; Jin-Hu, 2010; Guo et al., 2014b; Zhou et al., 2014) studied the problem of the synchronization stability of coupled oscillators in a continuous system and proposed a dynamic network consisting of N identical vibrators whose dynamic equation is:

(5) $$\begin{array}{l} \dot{x_{i}}=f\left(x_{i}\right)-c\overset{N}{\underset{j=1}{\sum }}{\text{l}}_{ij}H\left(x_{j}\right)\;,i=1,2,\;\ldots \ldots N \end{array}$$

where $x_{i}={\left(x_{i1},x_{i2},\ldots ,x_{in}\right)}^{T}\in R^{N}$ are the node's state variable; **x**_**i**_ = **f**(**x**_**i**_) describes the state of a single node when there is no coupling; c is the strength of the brain network coupling that has been constructed; H is a node state variable indicating which variables are passed between the coupled nodes; **L** is the Laplacian matrix of the brain network; **l**_**ij**_ is the matrix element of L and contains the information of the network topology.

When the coupling matrix is a Laplacian matrix:If L is a positive semidefinite symmetric matrix and the row sum is 0, then the eigenvalue of L satisfies the following when the network remains connected:

①matrix L has only one eigenvalue with a multiplicity of 1 and its corresponding eigenvector is

(1,1,1,1 … …1)^*T*^

②The remaining N-1 eigenvalues of the matrix L are positive real numbers, that is: 0 = λ1 < λ2 ≤ λ3 ≤ λ4 ≤ ……. ≤ λN_°_

Definition 3:

When the coupling matrix L satisfies Definition 2, the synchronization ability of the network can be expressed by the spectral features of the coupling matrix L. According to the different situations of the synchronization area, a dynamic network (Definition 2) can be divided into two categories. One (type 1) is that the synchronization field of the network is semi-unbounded, and its synchronization ability passes through the minimum non-zero spectral feature λ2 of the corresponding Laplacian matrix L. The larger the value of λ2, the stronger the synchronization ability. The other type (type 2) is that the synchronization domain of the network is bounded, and its synchronization capability can be characterized by the ratio R of the maximum non-zero spectral characteristics of the corresponding Laplacian matrix L to the smallest non-zero spectral features. The smaller the value of R, the stronger the synchronization capability.

Note:

(6) $$\begin{array}{l} R=\lambda \text{N}/\lambda 2 \end{array}$$

**Figure 3:**
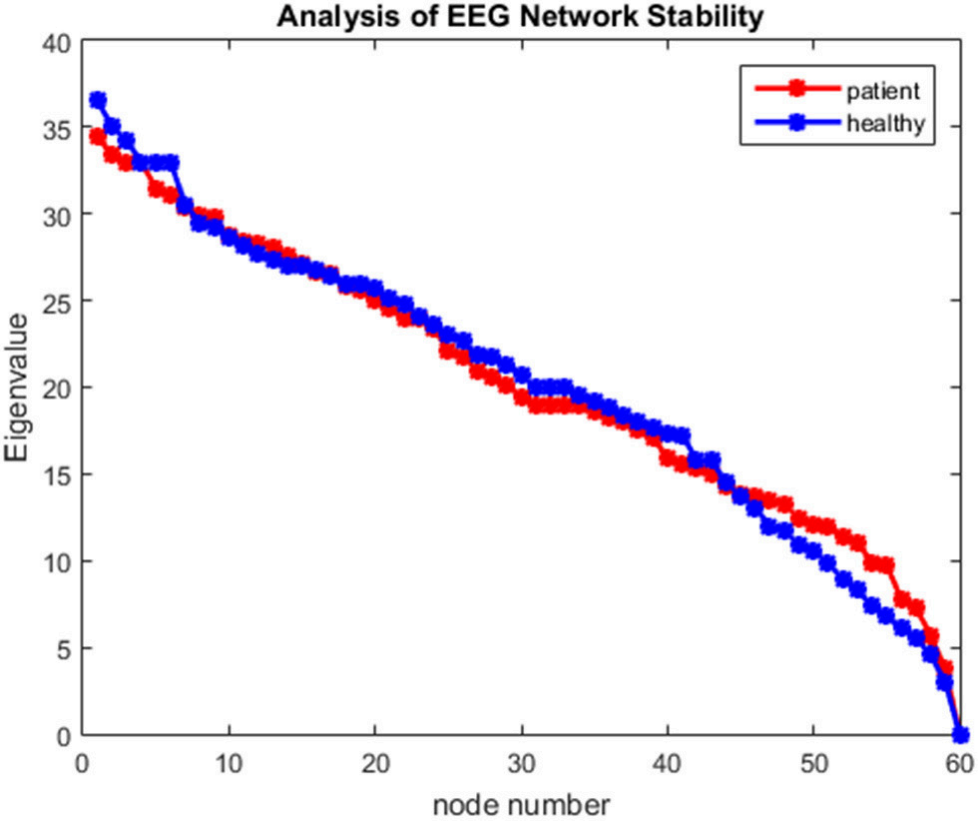
EEG Working memory data Laplacian eigenvalue spectrum (descending order) diagram.

Proof:

Construct a brain network of experimental datasets and compute the spectral features of the L-matrix. The experimental results shown in Figure 3 show that the spectral features of the brain network were all positive. The data verification used in this paper satisfies the synchronization criterion condition type 2. That is, the synchronization ability of the network can be measured by calculating the parameter R.

### Defining the Coupling Formula L^*^

Currently, the adjustment of the synchronization capability of complex network power systems (Wigand et al., 2015; Hongyue et al., 2017; Ruizhen et al., 2017) is mainly based on the network topology, adaptive synchronization control of dynamic equations, and network coupling methods. Considering the particularity of a brain network in the practical application process, that is, that the brain network structure is not easy to change and that the dynamic system is more complex and difficult to control, we here propose a method based on the fusion of traditional features and spectral features to achieve the ability of brain network synchronization for patients. In theory, adjusting a disease brain network so that it is synchronized with that of normal people could be a way to treat the disease.

The definition of the T&S(Traditional and Spectral)coupling matrix L^*^equationĩs:

(7) $$\begin{array}{l} L^{*}=L*G \end{array}$$

where matrix L is the original Laplacian matrix of the complex network dynamics equation, which represents the network spectrum characteristics. Matrix G is the distinctive feature of the extracted brain network.

Formula (7) can be written as formula (8):

(8) $$\begin{array}{l} {{l_{ij}^{*}=l_{ij}^{*}g}_{i}}^{aa}*{g_{j}}^{bb} \end{array}$$

Where ∀ aa, bbϵ R, we can adjust the parameters aa, bb to enhance or weaken the synchronization ability of the network. The combinations of parameters aa, bb are: ① parameters aa, bb are both positive; ② parameters aa, bb are one positive and one negative; ③ parameters aa, bb are both negative.

Define the T&S coupling matrix L^*^ [Equation (7)], If the T&S coupling matrix L^*^ satisfies definition 3 of section Synchronization Criteria,

which is:

L^*^ is a positive semidefinite symmetric matrix;

L^*^ line sum is 0;

Then the synchronization capability of the brain network is the ratio R of the spectral features of the T&S coupling matrix L^*^.

Mathematical proof:

① If the matrix L^*^ is a real symmetric matrix know all eigenvalues of L^*^ are real numbers;

② If the matrix L^*^ is a positive semi-definite matrix know all eigenvalues of L^*^ are positive or 0;

③ The following only proves that the matrix L^*^ satisfies the row sum to 0;

Proof:

Write formula (7) as a matrix:

(9) $$\begin{array}{l} L^{*}=L{*G^{aa}*G}^{bb} \end{array}$$

Where

*G* = *diag* {*k*_1_, *k*_2_…..*k*_*N*_} is a diagonal matrix composed of a distinctive feature of the brain network.

The sum of all elements in the i-th row of matrix L^*^ is:

(10) $$\begin{array}{l} L_{J}*=\sum_{i=1,j\neq i}^{N}L_{ij}g_{i}^{aa}*g_{j}^{bb}=\left(\sum_{i=1,j\neq i}^{N}L_{ij}g_{i}^{aa}\right)*g_{j}^{bb}\\ =\left(\sum_{i=1,j\neq i}^{N}L_{ij}g_{i}^{aa}-\left(\sum_{i=1,i\neq j}^{N}L_{ij}g_{j}^{-aa}g_{i}^{aa}g_{j}^{aa}\right)\right)*g_{j}^{bb}\\ \;=\sum_{i=1,i\neq j}^{N}L_{ij}g_{i}^{aa}g_{j}^{bb}-\sum_{i=1,i\neq j}^{N}L_{ij}g_{i}^{aa}g_{j}^{bb}=0 \end{array}$$

Through mathematics and experiments (Figure 3) prove: When the network remains connected, the spectral features of L^*^satisfy: (1) The matrix L^*^has only one eigenvalue with a multiplier of 1 and its corresponding eigenvector (1,1,1,1, … … 1)^*T*^; (2) The remaining N-1 eigenvalues of the matrix L^*^are positive real numbers, that is: 0 = λ1 < λ2 ≤ λ3 ≤ λ4 ≤ ……. ≤ λN.

## Experimental Results and Analysis

### Experiment 1:Brain-Network Significant Difference Features and Node Extraction

Experiment 1 investigated the encoding stage.

①Using the method of feature extraction described in section Brain-Network Feature Extraction, the distinctive features obtained in the first stage extraction included: assortativity (depending on the trend of nodes in the network, it can be divided into an assortative or disassortative network. Assortative means that a node tends to be connected to its similar node; otherwise, the network is said to be disassortative), mean-clustering-coefficient, transitivity (transitivity is the ratio of “triangles to triplets” in the network), global efficiency, modulus, and mean path length (in the current study, we reanalyzed EEG data from our previous publications Liting et al., 2017; Yuchi et al., 2017).

②We calculated the Pearson correlation coefficient of the features extracted from the first stage and the second stage and identified the features that were strongly correlated. We randomly selected 10 normal subjects and 10 patients with 7 significant differences in characteristics and used their network spectral characteristics (R) to calculate the Pearson correlation coefficients (Tables 2, 3). Table 1 shows the nodes that differed significantly between the patient and healthy controls, as identified using the KS test.

**Table 1:** Extracted nodes that showed differences in encoding/alpha/sparsity 34%.

**ELECTRODE NUMBER—ELECTRODE NAME**		
59-POZ	3-FP2	51-PO3
58-P2	60-P1	29-Pz
36-AF4	57-PO4	31-FPz
16-Cz	22-TP8	17-CPz
15-FC4	27-O2	28-O1
35-AF8	43-FC6	5-FC3
9-F4	10-Fz	11-FCz
13-TP7	14-FT8	32-AF3

**Table 2:** The average Pearson correlation coefficient for the 10 healthy controls.

**Normal/Encoding/Alpha**	**NO.1**	**NO.2**	**NO.3**	**NO.4**	**NO.5**	**NO.6**	**NO.7**	**NO.8**	**NO.9**	**NO.10**	**AVE**
Assortativity	0.59	0.20	0.57	0.237	0.571	0.059	0.602	0.326	0.549	0.152	0.385
**Mean-Clustering-Coef**	**0.79**	**0.531**	**0.567**	**0.259**	**0.697**	**0.834**	**0.824**	**0.595**	**0.718**	**0.588**	**0.6105**
**Transitivity**	**0.82**	**0.546**	**0.566**	**0.595**	**0.701**	**0.703**	**0.653**	**0.666**	**0.544**	**0.795**	**0.6588**
**Global-Efficiency**	**−0.769**	**−0.430**	**−0.554**	**−0.708**	**−0.718**	**−0.787**	**−0.787**	**−0.572**	**−0.892**	**−0.852**	**−0.7069**
Modularity	−0.234	0.163	−0.378	−0.337	0.369	0.063	0.549	−0.405	0.114	−0.484	−0.0579
**Mean-Path-Length**	**0.774**	**0.530**	**0.754**	**0.717**	**0.709**	**0.797**	**0.789**	**0.633**	**0.899**	**0.906**	**0.774**

*Bold indicates that the conclusion is drawn from the bold part*.

**Table 3:** The average Pearson correlation coefficient for the 10 patients' group.

**Patient/Encoding/Alpha**	**NO.1**	**NO.2**	**NO.3**	**NO.4**	**NO.5**	**NO.6**	**NO.7**	**NO.8**	**NO.9**	**NO.10**	**AVE**
Assortativity	0.25	0.32	0.29	0.275	0.683	0.847	0.314	0.383	0.545	0.604	0.4522
**Mean-Clustering-Coef**	**0.59**	**0.814**	**0.683**	**0.745**	**0.773**	**0.818**	**0.844**	**0.819**	**0.545**	**0.668**	**0.730**
**Transitivity**	**0.57**	**0.667**	**0.581**	**0.551**	**0.774**	**0.804**	**0.851**	**0.823**	**0.716**	**0.703**	**0.65883**
**Global-Efficiency**	**−0.735**	**−0.801**	**−0.530**	**−0.582**	**−0.804**	**−0.898**	**−0.841**	**−0.830**	**−0.885**	**−0.829**	**−0.7736**
Modularity	−0.285	0.092	−0.379	−0.119	−0.154	−0.062	−0.551	0.486	−0.150	−0.174	−0.1295
**Mean-Path-Length**	**0.667**	**0.802**	**0.635**	**0.709**	**0.804**	**0.797**	**0.848**	**0.839**	**0.902**	**0.826**	**0.667**

*Bold indicates that the conclusion is drawn from the bold part*.

Tables 2, 3 show that there was a strong positive correlation between the synchronization of the brain network and the mean-clustering-coefficient, transitivity, mean path length, and node degree and that there was a strong negative correlation with global efficiency.

The criteria for evaluating the Pearson correlation coefficient are: (1) 0.8–1.0 means that the two are highly correlated; (2) 0.6–0.8 means that the two are significantly correlated; (3) 0.4–0.6 means that the two are mildly correlated (4) 0.2–0.4 means that the two are weakly related; (5) 0.0–0.2 means that the two are very weakly related.

### Experiment 2:Brain-Network Synchronization Stability Analysis

#### Differences in Synchronous Processing

**Figure 4:**
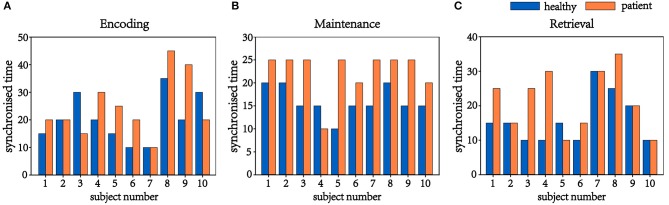
Time of initial synchronization in the healthy controls and the patients. **(A)** is the encoding stage, **(B)** is the maintenance stage, and **(C)** is the retrieval stage.

In this process, 20 brain networks were constructed for each subject's 20 trial EEG signals and were used to extract the synchronization features (R) of the brain network's spectral features. The patients and healthy controls could be represented by their synchronization features and by the time required to reach the initial synchronization. The process by which brain synchronization occurs during memory processing differs between healthy subjects and patients. Table 4 is the range of changes in the mean values of the synchronization characteristics for the 34 patients and 34 healthy controls in the encoding, maintenance, and retrieval stages; Figure 4 is the initial synchronization of the time chart for the 10 patients and 10 healthy controls who were randomly selected during the encoding, maintenance, and retrieval stages; Table 5 shows the mean and variance at the initial synchronization in Figure 4.

**Table 4:** Comparison of synchronization differences between healthy controls and patients.

**NORMAL GROUP AND PATIENT GROUP R-MEAN**		
	**Normal**	**Patient**
Encoding	**5–19**	**5–8**
Maintenance	5–13	5–8
Retrieval	5–8	5–8

*Bold indicates that the conclusion is drawn from the bold part*.

**Table 5:** Time to initial synchronization in patients and healthy controls.

**Subject**	**Stage/band** **(alpha)**	**Average** **value (ms)**	**Standard** **deviation (ms)**
Patients	Encoding	27	9.798
	Maintenance	26.5	10.259
	Retrieval	22	7.810
Normal	Encoding	18	6.782
	Maintenance	21	5.385
	Retrieval	15.5	6.874

Table 4 shows that there was a significant difference in the synchronization between the patients and the healthy controls in the encoding phase and that the patient's synchronization ability was stronger than that of the normal subjects. This may be because patients have cognitive impairments in memory and their thinking and speech are often confused. Therefore, they showed considerable differences from the healthy controls in the encoding phase of working memory. An analysis of Table 5 shows that the normal subjects achieved synchronization earlier than the patients.

#### Determining Significant Differences in the Synchronization Stability of Area S

##### A. Discovering-significant-differences-in-area-s

**Figure 5:**
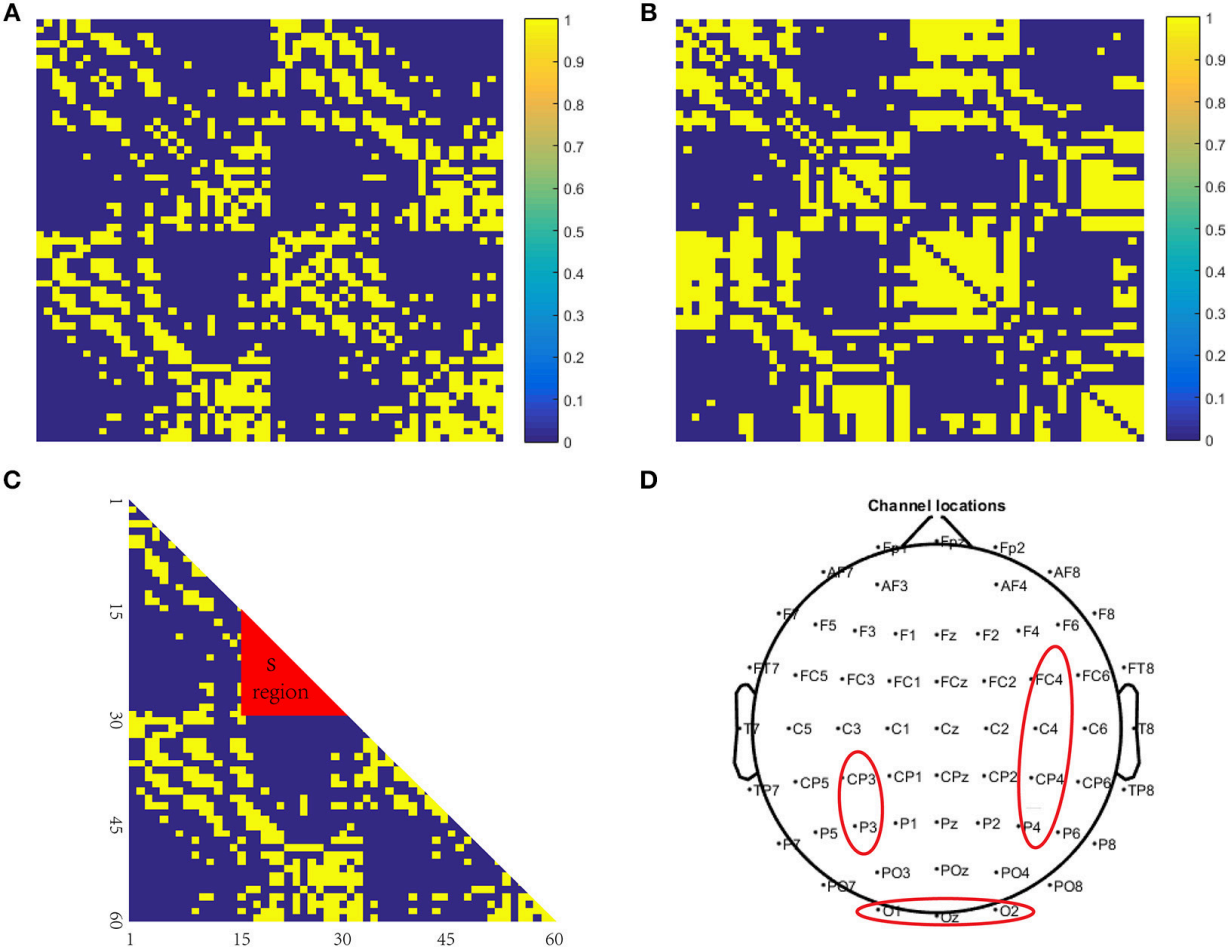
Brain network structure. **(A)** is a binary matrix constructed using the healthy controls; **(B)** is a binary matrix constructed using the patient subjects; **(C)** is the difference significant area S, a mathematical representation of **(A,B,D)** is a brain electrode position diagram corresponding to a **(C)** S region in the brain map.

The PLV binary matrix corresponding to 48,960 brain networks constructed using data from the patients and the healthy controls is shown in Figures 5A–C shows the difference significant area, S, a mathematical representation of the difference between 5a and 5b; Figure 5D is a brain electrode position diagram corresponding to a 5c S region in the brain map.

Comparing the differences (Figures 5A,B) we found that the significant differences in the brain network between normal subjects and patients were located in the S region (Figure 5C). The specific manifestations were as follows: The normal controls' S-area connections were tightly organized and the patients' S-area connections were sparsely disordered. This is likely because psychiatric (specifically schizophrenic in this study) patients have cognitive impairments in memory, and their thinking and speech are often confused. Therefore, in memory processing, the connections between the brain regions of the brains of the psychiatric patients were disorganized, but the brain connections of the normal controls showed obvious signs of organization.

From the above analysis, we concluded that the area that was significantly different between the patients and the healthy controls was region S, so the corresponding brain area was primarily located in the occipital lobe.

##### B. Regional-s-synchronization-stability-analysis

**Figure 6:**
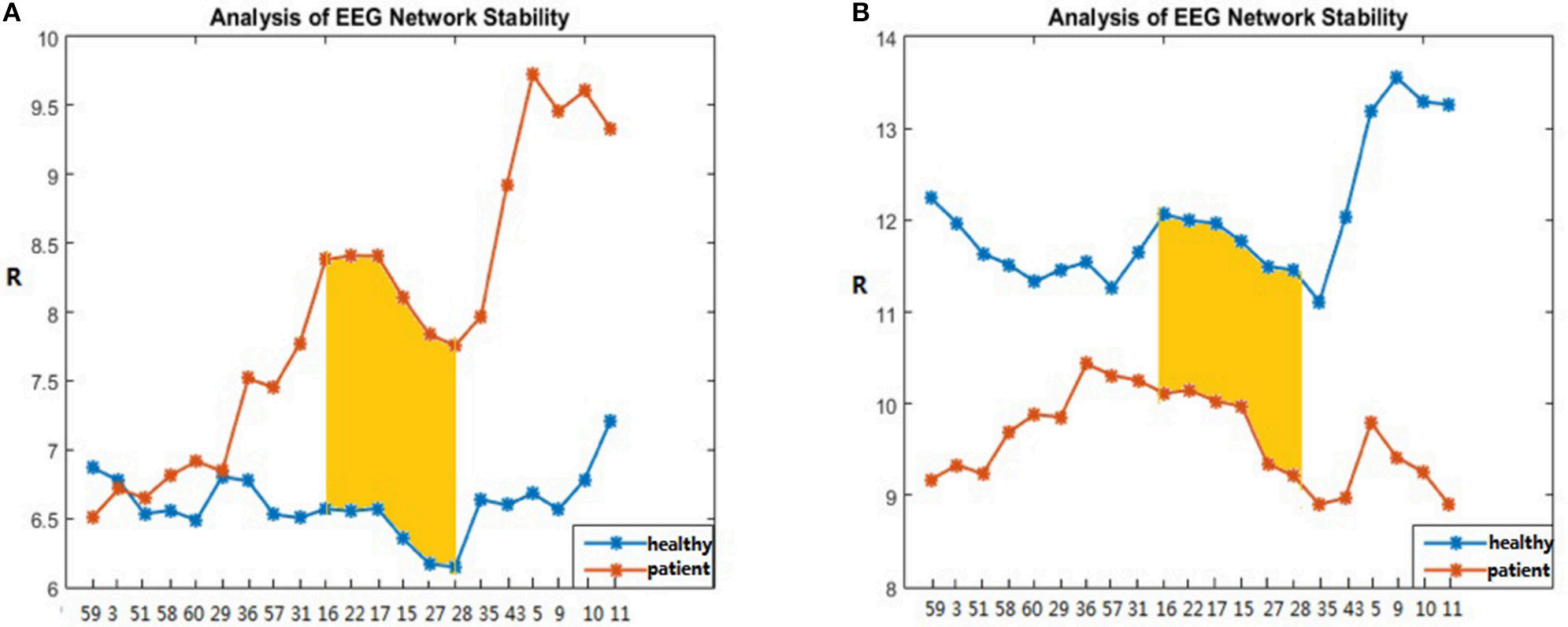
R-value changes with the coupling of the S area in patients and healthy controls. The patient subject graphed here was #6 and the healthy control subject was #4 in **(A)**, and the patient subject graphed in **(B)** was #4 and the healthy control subject was #6.

Using the information about the significant difference node (Table 1) extracted in Experiment1: Brain-Network Significant Difference Features and Node Extraction the coupling between a certain node in the brain network and the other nodes was removed, and the synchronous characteristics R of brain networks that were randomly selected from the patients and healthy controls (if the other subject conditions were kept constant) were the same. The subjects were compared based on the order of magnitude (Figure 6). In Figure 6, the abscissa represents the decoupling node number (the electrode corresponding to the node number in Table 6), and the ordinate represents the synchronization eigenvalue of the brain network after decoupling.

**Table 6:** EEG signal 64 electrode name numbering table.

**Ele-No**.	**EN**	**Ele-No**.	**EN**	**Ele-No**.	**EN**	**Ele-No**.	**EN**	**Ele-No**.	**EN**
1	Fp1	14	FT8	27	O2	40	F2	53	CP6
2	F7	15	FC4	28	O1	41	FC1	54	PO8
3	Fp2	16	Cz	29	Pz	42	C5	55	P6
4	F3	17	CPz	30	Oz	43	FC6	56	CP2
5	FC3	18	CP3	31	Fpz	44	FC2	57	PO4
6	FT7	19	P3	32	AF3	45	C2	58	P2
7	T7	20	P7	33	AF7	46	C1	59	POz
8	F8	21	T8	34	F5	47	CP1	60	P1
9	F4	22	TP8	35	AF8	48	CP5	61	VEOU
10	Fz	23	C4	36	AF4	49	P5	62	VEOL
11	FCz	24	P8	37	F1	50	C6	63	HEOL
12	C3	25	CP4	38	FC5	51	PO3	64	HEOR
13	TP7	26	P4	39	F6	52	PO7		

Figure 6 shows that, in the encoding stage, when node numbers 59, 3, 51, 58, 60, …, 9, 10, 11 were successively removed, the trends of the patients and the healthy controls in the Figure 6A were similar to those in Figure 6B. Particularly when the node numbers 16, 22, 17, 15, 27, 28 were sequentially removed, the synchronicity between the patient and the normal person increased. In addition, the node numbers 16, 22, 17, 15, 27, 28 all belong to the red area S, and the ability to remove these nodes one at a time was enhanced. The data shown in Figure 6 also indicate that, as the density of the red zone S edge decreased, the synchronization ability was stronger, and as the edge density increased, the worse the synchronization ability became (Figures 5A,B as healthy controls).

**Figure 7:**
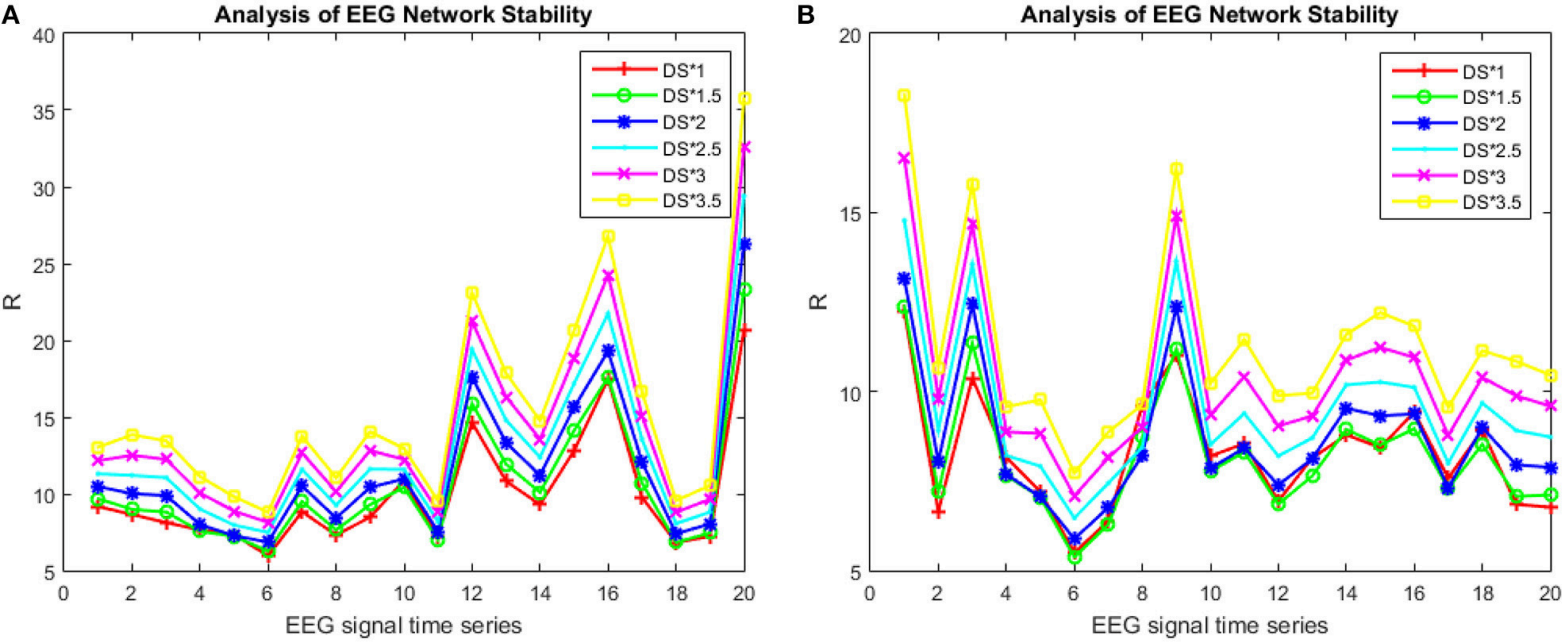
*R*-value changes with the strength of the S area in patients and healthy controls. The patient subject graphed here was #6 in **(A)** and the healthy control subject was #4 in **(B)**.

Without changing the coupling relationship of the red region S (Figure 5C), the effect of the influence on the synchronization ability of the brain network was observed by enhancing the edge strength of the red region S. Figures 7A,B show the changes in the synchronization ability of the red region S edge strengths of the 20 brain networks of a specific patient and a specific individual from the healthy controls from 1, 1.5…3.5 times (In the encoding phase, we chose a period of consecutive 100 s for each participant, constructing a brain network every 5 s).

It can be seen from Figure 7 that for the same edge strength, the change in the synchronization ability between the patients and the healthy controls exhibited opposite trends and tended to be uniform; the strength of the internal bridging of S is more obvious. However, if the edge strength was too great, it simultaneously reduced the ability of the healthy subjects' network and the patients' network to synchronize.

### Experiment 3: Synchronization Stability and Brain-Network Adjustment

From the analysis of the differences in the synchronization ability of the brain network between the patients and healthy controls in section Experiment 2:Brain-Network synchronization stability analysis, we found that the two groups differed considerably in their brains' synchronization ability and that these differences are concentrated in the local area S. That is, the smaller the S edge density, the stronger the synchronization ability. To achieve the goal of curing disease, this section primarily discusses the theory of complex network synchronization control to explore ways in which a patient's brain network could be given a certain “treatment” that would make the patient's synchronization ability consistent with the synchronization ability of normal people.

This section proposes a method based on the fusion of traditional features and spectral features to achieve the adjustment of the patient's brain network synchronization ability, so that its synchronization ability would be consistent with normal subjects, theoretically achieving the purpose of treatment of diseases. Applying the T&S coupling formula defined in section Defining the Coupling Formula L^*^, the patient's brain network synchronization ability could be adjusted by selecting appropriate values of the T&S coupling matrix L^*^ parameters aa or bb. The feasibility and validity of the method were verified by specific data.

#### Coupling Matrix L^∧*^Parameter Selection

**Figure 8:**
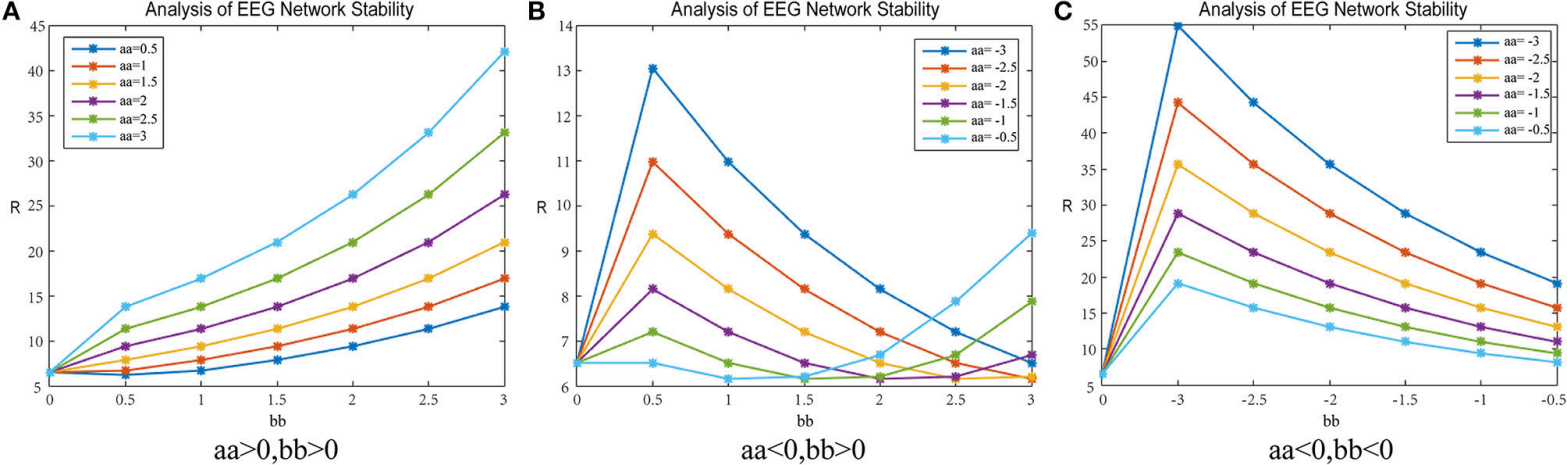
*R*-value changes in a patient subject. Matrix G represents mean-clustering-coef and the data are from patient #6. **(A)** shows the parameters aa, bb are positive. **(B)** shows the parameter aa is negative, bb is positive. **(C)** shows the parameter aa, bb are negative.

**Figure 9:**
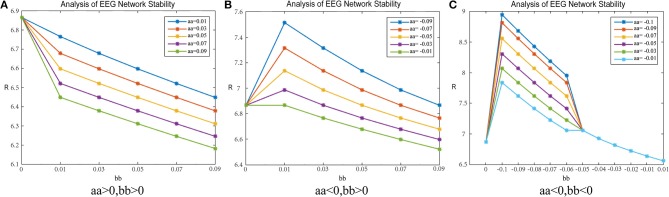
*R*-value changes in a healthy subject. Matrix G represents mean-path-length and the data are from patient #6. **(A)** shows the parameters aa, bb are positive. **(B)** shows the parameter aa is negative, bb is positive. **(C)** shows the parameter aa, bb are negative.

The experiment investigated the patients' brain networks. Based on the results of the Pearson correlation coefficient calculation in section Experiment1:Brain-Network Significant Difference Features and Node Extraction, the clustering coefficient of the matrix G and the mean path length were used to adjust the brain's synchronization ability. Figure 8 shows the change in the synchronization ability of the patient's brain network when the matrix G takes the clustering coefficient; Figure 9 shows the synchronization stability of the brain network when the matrix G takes the path length. The abscissa indicates the value of the parameter bb, and the ordinate indicates the corresponding brain network synchronization feature value. The parameters chosen in the experiment were arbitrary and have no practical meaning. Other values could also be selected.

##### Adjustment-A

Figure 8A shows the result when the parameters aa and bb are both positive; 8b shows the result when parameter aa is negative and bb is positive; 8c shows the result when the parameters aa and bb are both negative.

In Figure 8A, when the parameters aa and bb are positive numbers, the results are as follows: When parameter bb is fixed, the network synchronization capability is proportional to the value of parameter aa; when parameter aa is constant, the synchronization capability of the network has an anti-proportional relationship to the value of parameter bb.

In Figure 8B, when parameter aa is negative and bb is positive, the network synchronization capability changes are more complex. When parameter aa < −1.5, the trend of the network synchronization capability is exactly the same as that in graph c. When the parameter aa> −1.5, the synchronization trend of the network is exactly the same as that in graph a.

In Figure 8C, when the parameters aa and bb are both negative numbers, the results are as follows: When parameter bb is fixed, the network synchronization ability is inversely proportional to the value of parameter aa. When parameter aa is constant, the network synchronization ability increases with parameter bb and then decreases, and the network synchronization ability gradually changes to become consistent with the original synchronization capabilities.

##### Adjustment-B

Figure 9A shows the results when the parameters aa and bb are both positive; 9b shows the results when parameter aa is negative and bb is positive; 9c shows the results when parameters aa and bb are both negative.

In Figure 9A, when the parameters aa and bb are both positive, the results are as follows: When parameter bb is fixed, the network synchronization capability is directly proportional to the value of parameter aa; when parameter aa is fixed, the synchronization capability of the network is directly proportional to the value of parameter bb. This shows that, when the coupling matrix selects the path length as the “weighted” mode, the network synchronization capability is proportional to the values of the parameters aa and bb.

In Figure 9B, when parameter aa is negative and bb is positive, the results are as follows: When parameter bb is fixed, the network synchronization capability is directly proportional to the value of parameter aa; when parameter aa is fixed, the relationship between the synchronization capability of the network and the value of the parameter bb is first weakened and then enhanced to match the initial synchronization ability. After weakening, the enhancement tends to be consistent with the original synchronization ability.

In Figure 9C, when parameters aa and bb are negative, the network synchronization capability changes are more complex. When parameter bb is fixed and bb < −0.05, the synchronization capability of the network is proportional to the value of parameter aa; when parameter bb is fixed and bb > −0.05, the network synchronization capability is enhanced and does not change with parameter aa; when parameter aa is constant, the synchronization capability of the network decreases with an increase in parameter bb, and then the enhancement gradually changes to become stronger than the original synchronization capability.

#### Adjusting the Patient Synchronization Stability

**Figure 10:**
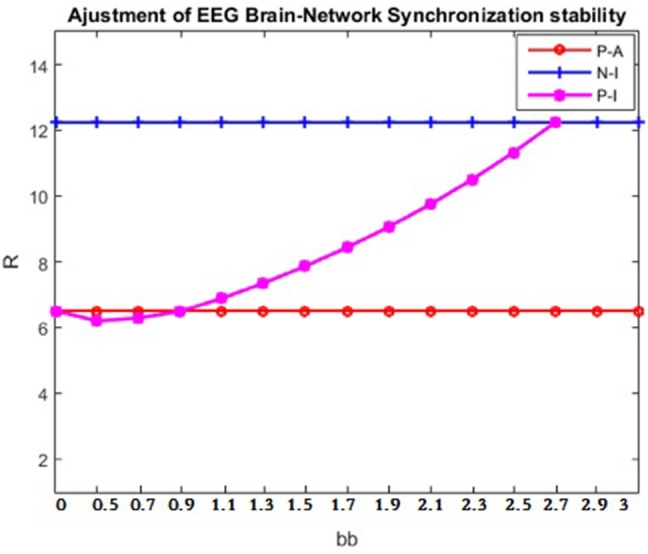
Adjustment of patient's brain- network synchronization ability. The data are from patient #6 and healthy control subject number #4. The abscissa is the value of parameter bb, and the ordinate is the corresponding brain network synchronization feature value. P-A indicates the synchronization ability of the patient after the brain network adjustment; P-I indicates the synchronization ability of the patient before the brain network adjustment; N-I indicates normal synchronization ability.

Based on the conclusions in Coupling Matrix L ^∧*^ Parameter Selection, the best values of parameters aa and bb could be selected to be applied to the patient, so that the synchronization ability of the brain network of a patient and the brain synchronization ability of a normal person would tend to be consistent. Next, the clinician would refer to the equivalent of Figure 8A to identify the parameter transformation and would select aa = 0.5. The patient's brain network synchronization ability would finally be adjusted using parameter bb (Figure 10). In Figure 10, it can be seen that when aa = 0.5 and bb = 2.5, the patient's synchronization ability should be consistent with that of a normal person. In this way, the goal of curing disease can theoretically be achieved.

## Limitations

The current study involved several limitations that should be considered. First, the effect of offline processing of EEG traces on brain network dynamics synchronization was not considered, so it is unclear how the synchronization differences obtained in an offline analysis of the EEG can pave a way for the treatment of unspecified neuropsychiatric diseases. In addition, EEG signals recorded from the scalp surface are generally highly correlated. Each channel is a linear mixture of concurrently active brain and non-brain electrical sources, whose activities are volume conducted to the scalp electrodes with broadly overlapping patterns (Nunez et al., 1997). Therefore, we used surface Laplacian transform methods to eliminate the mixed effect of volume conduction (Makeig et al., 1996; Jung et al., 2001; Delorme et al., 2012). Also, our study did not provide specific values for the parameters aa and bb, so the values of aa and bb were arbitrarily chosen. The purpose was only to verify the validity of the T&S coupling formula proposed in this study. As for its application in clinical trials, it may be necessary to correlate the aa and bb parameters with certain biological characteristics of the human body (such as blood flow). Consequently, we should pay more attention to these aspects and add related experiments in future research.

## Conclusion

In this study, a brain network was constructed based on complex network theory. The synchronization characteristics of the brain network were calculated using the spectral features of the brain network. The synchronization process characterizes the differences and changes in the brain network synchronization ability between a patient and healthy subjects during the process of making memories. Our experiments showed that the synchronization of aa differed significantly between the patients and the healthy controls and that this synchronization is concentrated in the S region. In addition, these experiments further indicated that the effect of S on the synchronization ability in this S region was that the density of the S region was smaller, and the synchronization ability was stronger. To achieve the purpose of treating patients, we proposed a method based on the fusion of traditional features and spectral features to achieve the adjustment of a patient's brain network synchronization ability. The KS test, SVM classification, and other methods were used to extract traditional features and nodes that showed significant differences; we designed a T&S coupling method that fuses traditional features with spectral features and selects the appropriate parameter values aa or bb to adjust the patient's brain network synchronization capabilities. The data validated the feasibility of the method and theoretically achieved the purpose of treating disease. This study has only theoretically explored the treatment of disease through algorithms and has not been clinically applied. In the future, we will try to explore animal (rat) susceptibility factors, clinical manifestations, skull characteristics, and prognosis in depth, and we hope to find feasible measures (such as physical therapy) that can adjust these features.

## Ethics Statement

All subjects were given written informed consent in accordance with the Declaration of Helsinki.

## Author Contributions

RY performed the experiment and completed the manuscript. XX, GY, HD, and PY provided suggestions for this study. HL provided the guidance throughout the study. HL had full access to all of the data in the study and takes responsibility for its integrity and the accuracy of the data analysis. All the authors have read through the manuscript, approved it for publication, and declared no conflict of interest.

### Conflict of Interest Statement

The authors declare that the research was conducted in the absence of any commercial or financial relationships that could be construed as a potential conflict of interest.
